# Estimating included animal species in mixed crude drugs derived from animals using massively parallel sequencing

**DOI:** 10.1038/s41598-021-85803-4

**Published:** 2021-03-18

**Authors:** Hiroaki Nakanishi, Katsumi Yoneyama, Masaaki Hara, Aya Takada, Kazuyuki Saito

**Affiliations:** 1 Department of Forensic Medicine, Juntendo University School of Medicine, 2-1-1, Hongo, Bunkyo-Ku, Tokyo, 113-8421 Japan; 2 Department of Forensic Medicine, Saitama Medical University, 38 Morohongo, Moroyama, Saitama 350-0495 Japan

**Keywords:** Next-generation sequencing, Targeted resequencing, Zoology

## Abstract

We developed a method that can detect each animal species of origin for crude drugs derived from multiple animal species based on massively parallel sequencing analysis of mitochondrial genes. The crude drugs derived from animals investigated in this study were Cervi Parvum Cornu and Trogopterorum feces, which are derived from a mix of different animal species, two chopped cicada sloughs, and two commercial Kampo drugs. The mitochondrial 12S rRNA, 16S rRNA, and cytochrome oxidase subunit I gene regions were amplified and sequenced using MiSeq. The ratios of haplotype to total number of sequences reads were calculated after sequence extraction and trimming. Haplotypes that exceeded the threshold were defined as positive haplotypes, which were compared with all available sequences using BLAST. In the Cervi Parvum Cornu and Trogopterorum feces samples, the haplotype ratios corresponded roughly to the mixture ratios, although there was a slight difference from mixture ratios depending on the gene examined. This method could also roughly estimate the compositions of chopped cicada sloughs and Kampo drugs. This analysis, whereby the sequences of several genes are elucidated, is better for identifying the included animal species. This method should be useful for quality control of crude drugs and Kampo drugs.

## Introduction

To maintain the quality of crude drugs, a stable supply of high-quality products is important. In Japan, the plant, animal, and mineral compositions of crude drugs listed in the Japanese Pharmacopoeia are regulated^1^. However, nonstandard or counterfeit crude drugs made using different plants or animals can be traded because crude drugs are often traded in locations far from where the plants were cultivated or the original animal habitat. Coghlan et al. tried to detect the species in many Kampo drugs using gene analysis and found animal DNA that was not clearly labeled on the packaging of 78% of the samples^2^. Therefore, identification of the species used to make crude drugs is essential for quality control.

Identification methods include sensory tests and morphological, macroscopic, and chemical examinations^3^. Molecular techniques have also been established to identify the origins of crude drugs^4^. Unlike morphological tests and other methods based on phenotypic characteristics, genotypic methods are not affected by environmental factors. Genotypic methods also have the advantage of not requiring specialized expertise; objective results are easy to obtain^3^.

To identify and classify animal species, several genes have been used, such as mitochondrial ribosomal RNA (rRNA)^5–7^, cytochrome oxidase subunit I (COI)^8,9^ and cytochrome b^10,11^. Many primers of rRNA that shorten amplicons to apply to degraded samples have been reported, although the genes of some species cannot be amplified^6,12^. The COI gene is widely used for DNA barcoding, although the amplicon by the universal primer is relatively long^13,14^. Many methods for identifying the animal origins of crude drugs by analyzing these genes have been reported^15–21^. However, these methods are based on Sanger sequencing, so are difficult to apply to mixtures containing nonstandard or counterfeit crude drugs, including those in powdered form.

Sequencing methods include Sanger sequencing and massively parallel sequencing (MPS). Sanger sequencing involves the electrophoretic separation of chain-termination products produced during individual sequencing reactions^22^. MPS involves repeated cycles of “polymerase-mediated nucleotide extensions” of many DNA fragments, for massively parallel sequencing^22^. In MPS analysis, if regarding each DNA fragment as haplotype, animal species would be identified by analyzing sequences of haplotypes, even within mixed samples. Moreover, the ratio of haplotypes that have the same sequence would show the ratio of animal species in mixed sample. This way of thinking is similar to 16S metagenomics^23^. In this study, we developed a method to detect each animal of origin of crude drugs derived from various animal species based on MPS analysis of several mitochondrial genes and applied this method to test real Kampo drugs. Especially, we evaluated whether 12S rRNA (12S), 16S rRNA (16S) and COI are suitable genes for MPS analysis.

## Results

### Analysis of single-source samples (S1–S4)

Table 1 summarizes the results of the analysis of single-source samples. The COI region was not amplified for either S3 or S4, so next-generation sequencing analysis was not performed. Animals that corresponded to each crude drug were detected for all samples (S1–S4). No positive haplotypes other than the expected animals were detected.

**Table 1:** Ratios of observed haplotypes and identification of the animal species of origin for S1–S4 via massively parallel sequencing (MPS). The top five haplotype ratios are shown. Only reads in which the proportion of all reads was at least 5% were analyzed by BLAST.

Sample	Haplotype	12S				16S				COI			
		Ratio against total reads (%)	Origin expected	Accession no	Match rate	Ratio against total reads (%)	Origin expected	Accession no	Match rate	Ratio against total reads (%)	Origin expected	Accession no	Match rate
S1	1st	91.47	*Cervus elaphus*	KX449334.1	109/109 (100%)	89.40	*Cervus elaphus*	KX449334.1	200/200 (100%)	85.74	*Cervus elaphus*	MF872247.1	225/225 (100%)
	2nd	0.45	–	–	–	0.22	–	–	–	2.59	–	–	–
	3rd	0.42	–	–	–	0.12	–	–	–	0.25	–	–	–
	4th	0.33	–	–	–	0.12	–	–	–	0.24	–	–	–
	5th	0.31	–	–	–	0.12	–	–	–	0.20	–	–	–
S2	1st	89.86	*Rangifer tarandus*	AB245426.1	109/109 (100%)	83.66	*Rangifer tarandus*	AB245426.1	200/200 (100%)	80.81	*Rangifer tarandus*	KJ205576.1	225/225 (100%)
	2nd	0.93	–	–	–	0.64	–	–	–	1.31	–	–	–
	3rd	0.60	–	–	–	0.46	–	–	–	1.25	–	–	–
	4th	0.52	–	–	–	0.18	–	–	–	1.14	–	–	–
	5th	0.45	–	–	–	0.17	–	–	–	1.13	–	–	–
S3	1st	89.86	*Trogopterus xanthipes*	AY227546.1	108/108 (100%)	73.76	*Trogopterus xanthipes*	AY227546.1	199/200 (99%)	Not amplified			
	2nd	0.86	–	–	–	1.79	–	–	–				
	3rd	0.56	–	–	–	0.99	–	–	–				
	4th	0.50	–	–	–	0.75	–	–	–				
	5th	0.36	–	–	–	0.68	–	–	–				
S4	1st	89.71	*Cavia porcellus*	MT017565.1	108/108 (100%)	76.59	*Cavia porcellus*	MT017565.1	200/200 (100%)	Not amplified			
	2nd	1.58	–	–	–	4.12	–	–	–				
	3rd	0.71	–	–	–	1.21	–	–	–				
	4th	0.48	–	–	–	0.85	–	–	–				
	5th	0.25	–	–	–	0.49	–	–	–				

### Analysis of mixed samples (M1–M10)

Table 2 (M1–M6) and Table 3 (M7–M10) summarize the results of the analyses of mixed samples. Only 12S and 16S were analyzed for M7–M10, because the COI region was not amplified in either S3 or S4. No positive haplotypes other than the expected animals were detected in any mixed sample.

**Table 2:** Ratios of observed haplotypes and identification of the animal species of origin for M1–M6 via MPS. The top five haplotype ratios are shown. Only reads in which the proportion of all reads was at least 5% were analyzed by BLAST. S1; Cervi Parvum Cornu derived from Cervus elaphus, S2; Cervi Parvum Cornu derived from Rangifer tarandus.

Sample	Mixture ratio (S1:S2)	Haplotype	12S		16S		COI	
			Ratio against total reads (%)	Origin expected	Ratio against total reads (%)	Origin expected	Ratio against total reads (%)	Origin expected
M1	1:1	1st	44.60	*Cervus elaphus*	38.48	*Rangifer tarandus*	54.22	*Cervus elaphus*
		2nd	41.13	*Rangifer tarandus*	33.96	*Cervus elaphus*	25.54	*Rangifer tarandus*
		3rd	2.77	–	2.96	–	2.22	–
		4th	0.43	–	2.61	–	2.03	–
		5th	0.31	–	1.81	–	1.30	–
M2	2:1	1st	56.73	*Cervus elaphus*	47.56	*Cervus elaphus*	61.47	*Cervus elaphus*
		2nd	30.20	*Rangifer tarandus*	26.35	*Rangifer tarandus*	16.75	*Rangifer tarandus*
		3rd	2.07	–	3.10	–	2.44	–
		4th	0.36	–	2.50	–	1.72	–
		5th	0.33	–	1.67	–	1.29	–
M3	4:1	1st	69.47	*Cervus elaphus*	59.93	*Cervus elaphus*	71.10	*Cervus elaphus*
		2nd	19.85	*Rangifer tarandus*	17.51	*Rangifer tarandus*	11.96	*Rangifer tarandus*
		3rd	1.28	–	2.32	–	2.90	–
		4th	0.31	–	1.87	–	1.01	–
		5th	0.30	–	1.27	–	0.62	–
M4	9:1	1st	78.72	*Cervus elaphus*	69.92	*Cervus elaphus*	77.94	*Cervus elaphus*
		2nd	10.41	*Rangifer tarandus*	9.40	*Rangifer tarandus*	5.75	*Rangifer tarandus*
		3rd	0.67	–	1.62	–	2.83	–
		4th	0.57	–	1.23	–	0.49	–
		5th	0.50	–	1.02	–	0.43	–
M5	19:1	1st	84.77	*Cervus elaphus*	78.70	*Cervus elaphus*	81.69	*Cervus elaphus*
		2nd	5.41	*Rangifer tarandus*	5.12	*Rangifer tarandus*	3.52	– (*Rangifer tarandus*)
		3rd	0.44	–	0.83	–	2.88	–
		4th	0.42	–	0.73	–	0.41	–
		5th	0.42	–	0.50	–	0.35	–
M6	39:1	1st	87.86	*Cervus elaphus*	84.80	*Cervus elaphus*	83.63	*Cervus elaphus*
		2nd	2.56	– (*Rangifer tarandus*)	1.85	– (*Rangifer tarandus*)	2.94	– (*Rangifer tarandus*)
		3rd	0.60	–	0.43	–	1.46	–
		4th	0.43	–	0.33	–	0.27	–
		5th	0.31	–	0.23	–	0.27	–

**Table 3:** Ratios of observed haplotypes and identification of the animal species of origin for M7–M10 via MPS. Only reads in which the proportion of all reads was at least 5% were analyzed by BLAST. S3; Trogopterorum feces derived from *Trogopterus xanthipes*, S4; Trogopterorum feces derived from *Cavia porcellus*.

Sample	Mixture ratio (S3:S4)	Haplotype	12S		16S	
			Ratio against total reads (%)	Origin expected	Ratio against total reads (%)	Origin expected
M7	1:1	1st	49.93	*Cavia porcellus*	39.86	*Trogopterus xanthipes*
		2nd	36.02	*Trogopterus xanthipes*	35.94	*Cavia porcellus*
		3rd	0.86	–	1.25	–
		4th	0.66	–	1.12	–
		5th	0.62	–	1.09	–
M8	2:1	1st	48.78	*Trogopterus xanthipes*	47.67	*Trogopterus xanthipes*
		2nd	36.34	*Cavia porcellus*	26.50	*Cavia porcellus*
		3rd	1.14	–	1.37	–
		4th	0.80	–	1.27	–
		5th	0.72	–	1.10	–
M9	4:1	1st	58.26	*Trogopterus xanthipes*	56.79	*Trogopterus xanthipes*
		2nd	27.61	*Cavia porcellus*	16.85	*Cavia porcellus*
		3rd	0.92	–	1.95	–
		4th	0.70	–	0.92	–
		5th	0.64	–	0.76	–
M10	9:1	1st	71.56	*Trogopterus xanthipes*	67.48	*Trogopterus xanthipes*
		2nd	16.10	*Cavia porcellus*	7.82	*Cavia porcellus*
		3rd	0.75	–	1.72	–
		4th	0.64	–	1.07	–
		5th	0.54	–	0.72	–

For M1–M6, the haplotype ratios observed corresponded roughly to the mixture ratios for 12S and 16S, whereas the observed haplotype ratios did not reflect the mixture ratios for COI well because more of the *Cervus elaphus* gene was detected than expected. In terms of the detection limit for minor contents, our results indicate that minor contents can be detected from mixtures of two animals if the mixtures contain at least 19:1 mtDNA (M5) based on 16S.

For M7–M10, the observed haplotype ratios corresponded roughly to the mixture ratios for 16S, whereas the observed haplotype ratios did not reflect the mixture ratios for 12S well because more of the *Cavia porcellus* gene was detected than expected.

### Analysis of the test samples (C1, C2, Kampo-A, and Kampo-B)

The results of the analysis of the test samples are summarized in Table 4 (C1 and C2) and Table 5 (Kampo-A and Kampo-B).

**Table 4:** Ratios of observed haplotypes and identification of the animal species of origin for C1 and C2 via MPS. Only reads in which the proportion of all reads was at least 5% were analyzed by BLAST.

Sample	Haplotype	12S-C			
		Ratio against total reads (%)	Origin expected	Accession no	Match rate
C1	1st	80.57	*Meimuna iwasakii*	MG737724.1	205/222 (92%)
			*Meimuna oshimensis*	MG737727.1	204/221 (92%)
	2nd	2.62	–	–	–
	3rd	1.00	–	–	–
	4th	0.87	–	–	–
	5th	0.40	–	–	–
C2	1st	54.02	*Meimuna iwasakii*	MG737724.1	205/222 (92%)
			*Meimuna oshimensis*	MG737727.1	204/221 (92%)
	2nd	11.07	*Oncotympana maculaticollis*	JQ910987.1	204/217 (94%)
	3rd	8.77	*Meimuna iwasakii*	MG737724.1	204/222 (92%)
			*Meimuna oshimensis*	MG737727.1	203/221 (92%)
	4th	1.78	–	–	–
	5th	1.69	–	–	–

**Table 5:** Ratios of observed haplotypes and identification of the animal species of origin for Kampo-A and -B via MPS. Only results in which the proportion of all reads was at least 1% are shown.

Sample	Haplotype	12S				16S				COI			
		Ratio against total reads (%)	Origin expected	Accession no	Match rate	Ratio against total reads (%)	Origin expected	Accession no	Match rate	Ratio against total reads (%)	Origin expected	Accession no	Match rate
Kampo A	1st	69.22	*Cervus elaphus*	KX449334.1	109/109 (100%)	66.91	*Cervus elaphus*	KX449334.1	200/200 (100%)	30.71	*Cervus elaphus*	KX449334.1	225/225 (100%)
	2nd	5.28	*Cervus elaphus*	KP172593.1	109/109 (100%)	6.00	*Cervus nippon*	MH997432.1	200/200 (100%)	21.85	*Cervus elaphus*	MF872247.1	225/225 (100%)
	3rd	5.06	*Saiga tatarica*	MF497028.1	109/109 (100%)	3.87	*Saiga tatarica*	MF497028.1	200/200 (100%)	6.70	*Saiga tatarica*	JN632700.1	225/225 (100%)
	4th	–	–	–	–	–	–	–	–	2.91	*Cervus elaphus*	KJ025072.1	225/225 (100%)
	5th	–	–	–	–	–	–	–	–	2.75	*Cervus elaphus*	KX449334.1	223/225 (99%)
	6th	–	–	–	–	–	–	–	–	2.74	*Bufo gargarizans*	KY385799.1	225/225 (100%)
	7th	–	–	–	–	–	–	–	–	2.61	*Cervus nippon*	KF934184.1	225/225 (100%)
Kampo B	1st	24.05	*Cervus elaphus*	KP172593.1	109/109 (100%)	35.94	*Arctocephalus pusillus*	AM181018.1	200/200 (100%)	17.67	*Cervus elaphus*	KJ205555.1	224/225 (99%)
	2nd	22.74	*Arctocephalus pusillus*	AM181018.1	108/109 (99%)	10.57	*Cervus elaphus*	KP172593.1	200/200 (100%)	8.28	*Cervus elaphus*	KJ205555.1	225/225 (100%)
	3rd	14.30	*Arctocephalus pusillus*	AM181018.1	109/109 (100%)	6.42	*Phoca groenlandica*	MH198019.1	200/200 (100%)	7.75	*Arctocephalus pusillus*	AM181018.1	223/225 (99%)
	4th	9.31	*Phoca groenlandica*	MH198019.1	109/109 (100%)	2.57	*Cervus nippon*	JN389443.1	200/200 (100%)	5.70	*Cervus elaphus*	KX859259.1	225/225 (100%)
	5th	1.97	*Bos taurus*	MN714218.1	109/109 (100%)	1.62	*Bos taurus*	MN714218.1	200/200 (100%)	4.85	*Cervus elaphus*	MF872247.1	225/225 (100%)
	6th	1.85	*Cervus elaphus*	KP172593.1	108/109 (99%)	1.50	*Callorhinus ursinus*	MG916809.1	198/200 (99%)	3.05	*Cervus elaphus*	KP172593.1	225/225 (100%)
	7th	1.20	*Cervus elaphus*	KP172593.1	108/109 (99%)	1.40	*Cervus elaphus*	KP172593.1	199/200 (99%)	2.95	*Elaphe carinata*	MK064637.1	225/225 (100%)
	8th	–	–	–	–	1.38	*Cervus elaphus*	KP172593.1	193/193 (100%)	2.87	*Phoca groenlandica*	MH198023.1	225/225 (100%)
	9th	–	–	–	–	–	–	–	–	2.50	*Arctocephalus pusillus*	AM181018.1	222/225 (99%)
	10th	–	–	–	–	–	–	–	–	2.42	*Cervus elaphus*	NC_039923.1	225/225 (100%)
	11th	–	–	–	–	–	–	–	–	1.50	*Arctocephalus pusillus*	AM181018.1	222/225 (99%)
	12th	–	–	–	–	–	–	–	–	1.47	*Arctocephalus pusillus*	AM181018.1	225/225 (100%)
	13th	–	–	–	–	–	–	–	–	1.37	*Arctocephalus pusillus*	AM181018.1	222/224 (99%)
	14th	–	–	–	–	–	–	–	–	1.13	*Cervus elaphus*	KX449334.1	225/225 (100%)

In the analysis of the chopped cicada sloughs (C1 and C2), although the match rates were lower than for other animals (92–94%), one genus (*Meimuna*) was detected in C1 and two genera (*Meimuna* and *Oncotympana*) were detected in C2. No positive haplotypes other than those from Cicadae were detected in either sample.

In Kampo-A, the 12S genes of animals used in Cervi Parvum Cornu, Bezoar Bovis, and Saigae tataricae Cornu were detected, as were the COI genes of animals used in Cervi Parvum Cornu, Bufonis Venenum, and Saigae tataricae Cornu. However, only the 16S genes of animals used in Cervi Parvum Cornu and Saigae tataricae Cornu were detected. In Kampo-B, the 12S, 16S, and COI genes of animals used in Phocae Testis et Penis, Cervi Parvum Cornu, and Bezoar Bovis were detected; in addition, the COI gene of the snake *Elaphe carinata* was detected (COI haplotype 7th, Table 5).

## Discussion

To develop a method to detect each animal species of origin using MPS, we first examined whether it is possible to identify animal species using pure samples (S1–S4). For each pure sample, only one positive haplotype was observed, so it was suggested that the threshold positive-haplotype ratio defined here (5%) is suitable for the analysis of animal-derived crude drugs. The COI genes of some animal species might not be amplified with the COI primers, because the COI gene was not amplified for Trogopterorum feces. In the mixed Cervi Parvum Cornu samples (M1–M6), the observed haplotype ratios corresponded roughly to the mixture ratios for 12S and 16S, whereas a slight difference from the mixture ratios for COI was observed. PCR amplification efficiency differs according to the affinity with the primer sequences^24^. Therefore, the difference in PCR amplification efficiency between *C. elaphus* and *R. tarandus* might be due to differences in their 12S and 16S primer region sequences (Supplementary Table S1). In the mixed Trogopterorum feces samples (M7–M10), the ratio for *Cavia porcellus* tended to be higher for 12S than for 16S. One base at the 5′-end of the 12S forward primer differs from the corresponding base in the *T. xanthipes* sequence, whereas both the forward and reverse primers for 12S match the *Cavia porcellus* sequence. This difference might have caused the difference in PCR amplification efficiency. Therefore, if certain animal species are suspected to be in a drug, it is better to use a specific primer set for those sequences. The results for the chopped cicada sloughs are a good example of why a primer set whose sequences match those of the suspected animal species should be used. For the chopped cicada sloughs, the 12S, 16S, and COI genes were not amplified by the primer sets used, so the 12S-C primer set, which amplifies cicada genes, was used. The results suggested that the compositions of cicada sloughs from different suppliers differed.

This method could be used to roughly estimate the compositions of Kampo drugs by setting the threshold positive-haplotype ratio to 1%. The 12S and 16S analyses of Kampo-A both detected *Cervus elaphus* and *Saiga tatarica*, whereas the COI analysis detected *Cervus* spp. (*Cervus elaphus* and *Cervus nippon*), *Saiga tatarica*, and *Bufo gargarizans*. No genes were detected for *Bos taurus* from Bezoar Bovis or *Sus* species from Swine bile at the 1% level, although *Bos taurus* 12S was detected at a level of 0.94%. In Kampo-B, the 12S analysis detected *Arctocephalus pusillus*, *Cervus elaphus*, *Phoca groenlandica*, and *Bos taurus*, whereas the 16S analysis detected *Arctocephalus pusillus*, *Cervus* sp. (*C*. *elaphus* and *C*. *nippon*), *Phoca groenlandica*, *Callorhinus ursinus*, and *Bos taurus*, and the COI analysis detected *Cervus elaphus*, *Arctocephalus pusillus*, *Elaphe carinata*, and *Phoca groenlandica*. No civet (Viverridae) genes were detected. Therefore, although the animal species detected depended on the gene regions examined, no animal species that were not described in the attached documents were detected in either Kampo-A or -B. However, not every animal species described in the attached documents was detected. Arulandhu et al. reported that the COI gene was the most effective DNA barcode marker for animal species identification in MPS analysis^25^. However, because the animal species detected in a Kampo drugs depended on the gene regions examined, it was suggested that sequences for several genes should be evaluated for identifying the included animal species. Moreover, using several types of primers in the same region could be effective. *E. carinata*, a member of the snake family Colubridae, was shown by analysis of Kampo-B to be an origin animal of Agkistrodon Japonicae. Generally, Agkistrodon Japonicae is believed to be derived from *Gloydius blomhoffii*. However, in Japan, Colubridae and *G. blomhoffii* are also accepted as origin animals of Agkistrodon Japonicae^26^. Therefore, it was suggested that *E. carinata* was used instead of *G. blomhoffii*.

In addition to 16S metagenome analysis, the MPS methods used to discriminate species or individuals can be applied in forensic science^27^, food quality control^28^, and other applications. Both the forensic science and food quality control applications were focused on single-species targets, humans in the former and tuna in the latter, so primer design was easy, and the mixture ratios detected should reflect the real mixture ratios. However, a crude drug or Kampo drugs may be derived from various animal species. In practice, we suggest that the included animal species should be detected via species-specific tests, such as quantitative PCR methods, after using the MPS method as a screening test.

## Methods

### Samples

Cervi Parvum Cornu derived from *Cervus elaphus* (S1) and Trogopterorum feces derived from *Trogopterus xanthipes* (S3) were supplied by Tochimoto Tenkaido (Osaka, Japan). As nonstandard crude drugs, we purchased Cervi Parvum Cornu derived from *Rangifer tarandus* (S2) and Trogopterorum feces derived from *Cavia porcellus* (S4) from a pharmacy in Japan. Two chopped cicada sloughs (C1 supplied by Tochimoto Tenkaido and C2 purchased from a pharmacy in Japan) and two commercial Kampo drugs (Kampo-A and Kampo-B) containing crude drugs derived from animals were used as test samples. Kampo-A includes Cervi Parvum Cornu, Bezoar Bovis, Bufonis Venenum, Saigae tataricae Cornu, and Swine bile, whereas Kampo-B includes Phocae Testis et Penis, Cervi Parvum Cornu, Bezoar Bovis, Agkistrodon Japonicae, and Civet.

### DNA extraction

Crude drugs (50 mg) and Kampo drugs (200 mg) were used for DNA extraction, which was performed in accordance with a previously described protocol^15,16^. Briefly, each sample was first powdered. Then, ≤ 50 mg of sample was treated in a tube with 50 μL of proteinase K (QIAGEN, Venlo, The Netherlands) and 200 μL of Buffer ATL (QIAGEN), and the mixture was incubated overnight at 56 °C. Next, 500 μL of tris-ethylenediaminetetraacetic acid (EDTA)-saturated phenol (pH 8.0) was added and the solution was mixed thoroughly. After centrifugation at 15,000 rpm for 10 min, the water layer was transferred to a new tube. Then, 200 μL of Buffer AL (QIAGEN) was added, and the mixture was incubated at 70 °C for 10 min followed by the addition of 200 μL of ethanol. Next, the DNA was purified using a QIAamp DNA Mini Kit (QIAGEN) according to the manufacturer's protocol. The DNA was subsequently eluted into 60 μL of Buffer EB (QIAGEN).

Extracted DNA was quantified using a Qubit dsDNA HS Assay Kit and the Qubit 3.0 fluorometer (Thermo Fisher Scientific, Waltham, MA, USA). Mixed samples (M1–M10) were prepared in various ratios after normalizing the copy number based on the results of real-time polymerase chain reaction (PCR). Real-time PCR was performed in 20-µL reaction mixtures containing 10 µL TB Green Premix Ex Taq II (Takara Bio, Otsu, Japan), 1.6 µL of each 10 µM oligonucleotide primer (primer set for 16S rRNA in this study), and 2 µL of template DNA. PCR amplifications were performed using the StepOnePlus Real-Time PCR System (Thermo Fisher Scientific) at 95 °C for 30 s, followed by 40 cycles of 95 °C for 5 s and 60 °C for 30 s.

### Library preparation and MPS

To amplify the mitochondrial 12S, 16S, and COI gene regions, primer sets were created based on universal primers^6–8^. A primer set for amplifying 12S in Cicadoidea, named 12S-C, was created^29^. The sequences of each primer set are shown in Table 6. PCR was performed in 20-μL reaction mixtures containing 10 μL KOD One PCR Master Mix (Toyobo, Osaka, Japan), 1 μL of 10 μM oligonucleotide primers (final concentration 0.5 μM each), and 1 μL of < 10 ng template DNA. PCR was performed using a SimpliAmp Thermal Cycler (Thermo Fisher Scientific) with the following program: 40 cycles of 98 °C for 10 s, 55 °C for 5 s, and 68 °C for 1 s. Following PCR cleanup using Sera-Mag Select (Cytiva, Sheffield, UK), libraries were prepared using a Nextera XT Index Kit (Illumina, San Diego, CA, USA) and KAPA HiFi HotStart ReadyMix (Kapa Biosystems, Wilmington, MA, USA), according to the manufacturers’ instructions. Following PCR cleanup using Sera-Mag Select, the libraries were quantified using a Qubit dsDNA HS Assay Kit and Qubit 3.0 fluorometer, and fragment size was evaluated using a DNA-1000 kit with MultiNA (Shimadzu, Kyoto, Japan). The libraries were normalized to 4 nM, pooled, and diluted to 8 pM for sequencing. Then, 480 µL of 8 pM pooled library solution and 120 µL of 8 pM PhiX control solution were mixed and sequenced (2 × 251 cycles) using the MiSeq Reagent Kit v2 (500 cycles; Illumina), following the manufacturer’s instructions.

**Table 6:** Primer sequences used in this study. Underlined bases are tag sequences.

Primer set	Direction	Sequence (5′–3′)	Reference
12S	Forward	TCGTCGGCAGCGTCAGATGTGTATAAGAGACAGCCCAAACTGGGATTAGATACC	^6^
	Reverse	GTCTCGTGGGCTCGGAGATGTGTATAAGAGACAGTACAGAACAGGCTCCTCTAG	
16S	Forward	TCGTCGGCAGCGTCAGATGTGTATAAGAGACAGGCCTGTTTACCAAAAACATCAC	^7^
	Reverse	GTCTCGTGGGCTCGGAGATGTGTATAAGAGACAGCTCCATAGGGTCTTCTCGTCTT	
COI	Forward	TCGTCGGCAGCGTCAGATGTGTATAAGAGACAGGGWACWGGWTGAACWGTWTAYCCYCC	^8^
	Reverse	GTCTCGTGGGCTCGGAGATGTGTATAAGAGACAGTANACYTCNGGRTGNCCRAARAAYCA	
12S-C	Forward	TCGTCGGCAGCGTCAGATGTGTATAAGAGACAGAAACTAGGATTAGATACCCTATTAT	^29^
	Reverse	GTCTCGTGGGCTCGGAGATGTGTATAAGAGACAGAAGAGCGACGGGCGATGTGT	

### Detecting animal species of origin

The fastq files in the forward read direction were used to analyze 12S, COI, and 12S-C, whereas files in the reverse read direction were used to analyze 16S. The fastq files were analyzed by referring to a protocol described in our previous study^27^. Briefly, the CLC Genomics Workbench 20 (QIAGEN) was used to perform sequence extraction and trimming. The analysis conditions were set as follows: trim using a quality score of 0.001, maximum number of ambiguous reads set to 0, automatic read-through adapter trimming was checked, forward and reverse primers were removed from each primer set, and the filter for length in each amplicon was set as the length of the read with the greatest number of reads by referring to the length distribution in the supplementary QC report^27^. Trimmed reads were exported as CSV files. The number of haplotypes was counted using Excel (Microsoft Corp., Redmond, WA, USA). Haplotype ratios were calculated by dividing the number of each haplotype by the total number of reads. Haplotypes exceeding the haplotype ratio threshold of 5% were defined as positive haplotypes^27^. For Kampo-A and Kampo-B, the haplotype ratio threshold was 1%. Positive haplotypes were compared with all available sequences using BLAST (www.ncbi.nlm.nih.gov/BLAST). The animal species with the top score in the BLAST analysis was defined as the species of origin for a given crude drug.

## Supplementary Information

Supplementary Table S1.
